# Supplementary material for: A Likelihood Approach for Real-Time Calibration of Stochastic Compartmental Epidemic Models
Source: PLoS Comput Biol. 2017 Jan 17;13(1):e1005257. doi: 10.1371/journal.pcbi.1005257 (PMC5240920; doi:10.1371/journal.pcbi.1005257)
Supplement: S1 File — (TAR.GZ) [file pcbi.1005257.s014.tar.gz › HSPH_Online-SI-Revision/EnKF07/n100-extreme/n100-extreme_EnKF_table-8.pdf]

1	R0	{2.76052, 16.3913}	{2.19224, 4.41812}	{{1.04895, 2.64581}, {3., 5.00733}}	{28.9141, 73.046}	inf	695.	14 507.8	{5075.75, 46 684.6}	1988.7	1.18576	newly	417.	15 386.8	{5296., 50 041.}	3589.88	1.38877	Reff	2.73137	0.337952	{0.0670542, 0.582889}	87.627	ar	92 090.	163 512.	{109 375., 194 820.}	80.439
2	R0	{2.18202, 15.1734}	{2.29028, 9.42099}	{{0.407928, 2.70444}, {3., 13.7169}}	{42.7964, 37.9111}	inf	2011.	4373.25	{2787.29, 2787.29}	118.257	0.193522	newly	1160.	2951.27	{1155., 3978.}	154.809	0.244862	Reff	2.10076	0.379863	{0.074383, 0.651592}	81.9179	ar	84 264.	177 213.	{170 680., 175 079.}	110.307
3	R0	{2.57891, 12.5971}	{2.56207, 5.43986}	{{1.81442, 1.28267}, {3., 12.8264}}	{19.9204, 58.0252}	inf	340.	23 494.4	{4862.87, 58 170.3}	6810.12	1.68207	newly	303.	26 777.6	{4905., 72 436.}	8737.49	1.74102	Reff	2.5648	0.616835	{0.255884, 0.984114}	75.95	ar	90 302.	146 836.	{74 329., 247 020.}	68.2539
4	R0	{2.5856, 9.28508}	{2.25084, 9.52932}	{{0.578755, 2.10869}, {3., 14.8368}}	{29.8245, 43.5673}	inf	8.	19 197.9	{4655.14, 44 107.1}	239 874.	3.22642	newly	20.	24 406.6	{3975., 71 205.}	121 933.	2.82307	Reff	2.58508	1.54485	{0.274785, 2.3807}	42.6965	ar	90 312.	119 304.	{50 812., 194 791.}	49.4682
5	R0	{2.4406, 12.1341}	{2.46177, 7.16293}	{{2.15969, 4.31837}, {3., 9.89802}}	{15.7287, 46.0646}	inf	184.	21 247.9	{6614.81, 41 153.5}	11 447.8	1.93218	newly	170.	23 063.3	{5720., 52 126.}	13 466.7	1.96395	Reff	2.43279	0.997444	{0.311337, 2.18556}	59.	ar	88 597.	150 691.	{83 367., 212 903.}	72.8212
6	R0	{2.72155, 15.4094}	{2.2113, 4.13903}	{{1.05501, 2.64581}, {3., 5.00842}}	{29.1843, 73.1396}	inf	411.	14 464.2	{6271.93, 25 771.8}	3420.97	1.41308	newly	302.	15 921.5	{5339., 42 734.}	5172.02	1.55699	Reff	2.7028	0.380208	{0.130057, 0.792607}	85.9328	ar	91 682.	164 217.	{96 552., 205 545.}	83.8662
7	R0	{2.63781, 14.3935}	{2.48219, 4.97999}	{{2.00525, 1.29414}, {3., 7.55091}}	{19.9047, 65.4011}	inf	448.	2070.64	{432.533, 2251.7}	367.567	0.472481	newly	307.	2831.7	{576., 4198.}	826.137	0.649598	Reff	2.61787	0.491392	{0.175111, 0.756968}	81.2293	ar	91 192.	179 569.	{167 199., 195 989.}	96.9127
8	R0	{2.82391, 16.104}	{2.12296, 3.8491}	{{1.05015, 2.64581}, {3., 5.00798}}	{30.4341, 76.0985}	inf	560.	14 412.9	{3854.54, 37 885.}	2478.99	1.24932	newly	385.	16 483.8	{3924., 48 977.}	4182.26	1.41299	Reff	2.79954	0.384141	{0.109413, 0.79299}	86.2784	ar	92 664.	162 155.	{108 770., 205 953.}	79.4025
9	R0	{2.80645, 14.6457}	{1.98875, 4.35454}	{{1.15721, 2.64575}, {3., 5.00909}}	{33.9842, 70.2675}	inf	226.	19 494.5	{8735.61, 57 629.2}	8526.	1.74529	newly	148.	20 998.3	{6609., 56 342.}	14 088.	1.93292	Reff	2.79573	0.393391	{0.0582673, 0.899396}	85.9289	ar	92 567.	175 703.	{101 836., 269 198.}	95.9977
10	R0	{1.86213, 5.4271}	{2.13243, 11.0307}	{{0.935179, 4.45594}, {3., 17.0591}}	{39.918, 114.535}	inf	32.	25 055.2	{5884.84, 45 194.1}	78 197.5	2.72792	newly	74.	21 941.9	{4884., 51 798.}	29 551.2	2.24438	Reff	1.8607	1.2946	{0.288631, 2.22216}	37.6335	ar	75 362.	125 485.	{70 946., 171 545.}	72.1661
11	R0	{2.27349, 14.6338}	{2.51159, 4.72139}	{{2.00433, 1.2647}, {3., 7.52886}}	{23.8028, 67.7364}	inf	1278.	2392.99	{761.276, 3067.85}	122.362	0.249068	newly	843.	3752.93	{691., 10 926.}	358.098	0.382697	Reff	2.22036	0.570888	{0.201312, 1.62094}	74.2885	ar	85 805.	183 830.	{166 976., 235 940.}	114.242
12	R0	{2.77261, 14.1894}	{1.99188, 4.1852}	{{0.0915857, 0.934337}, {3., 5.8302}}	{35.7037, 70.7339}	inf	206.	4565.35	{674.212, 8268.79}	2116.19	1.12428	newly	178.	4842.92	{667., 9256.}	2620.74	1.17259	Reff	2.76385	0.261228	{0.0134965, 0.451724}	90.5484	ar	92 508.	166 124.	{157 173., 183 856.}	84.53
13	R0	{2.65402, 19.3935}	{2.45387, 6.65729}	{{1.46424, 3.37802}, {3., 10.2923}}	{21.099, 65.6726}	inf	1354.	4387.1	{1493.23, 8781.37}	229.226	0.237008	newly	658.	5474.11	{742., 25 437.}	735.439	0.507368	Reff	2.59651	0.621393	{0.0211034, 1.46371}	76.0681	ar	91 184.	187 957.	{169 869., 244 989.}	106.129
14	R0	{1.75297, 4.45354}	{2.60255, 7.59453}	{{2.16031, 4.34706}, {3., 14.7083}}	{50.3454, 71.8431}	inf	18.	21 750.8	{6547.87, 35 882.9}	120 738.	2.93869	newly	29.	20 763.7	{5710., 50 338.}	71 499.1	2.68853	Reff	1.75187	0.908402	{0.334617, 2.13154}	55.3836	ar	71 263.	150 378.	{98 738., 218 592.}	113.627
15	R0	{1.99928, 7.1501}	{2.45789, 7.65022}	{{2.15982, 4.3325}, {3., 14.7107}}	{27.9254, 43.4208}	inf	97.	22 560.4	{6612.39, 41 099.2}	23 158.1	2.23454	newly	168.	23 469.1	{5745., 62 131.}	13 869.7	1.96963	Reff	1.99548	0.98915	{0.311558, 2.18544}	53.8439	ar	79 602.	152 606.	{84 997., 218 687.}	92.6135
16	R0	{2.67398, 10.3134}	{2.5877, 7.59435}	{{2.16025, 4.34365}, {3., 14.7091}}	{15.6627, 40.1559}	inf	44.	21 483.7	{5921.27, 35 307.1}	48 726.6	2.54273	newly	54.	20 550.8	{4639., 50 219.}	37 957.	2.40859	Reff	2.67219	0.894877	{0.329371, 2.13121}	66.5115	ar	91 289.	149 345.	{99 182., 218 507.}	68.3726
17	R0	{2.44634, 16.3226}	{2.42316, 6.60846}	{{1.46358, 3.37437}, {3., 10.2908}}	{25.2761, 59.5134}	inf	1288.	4327.73	{1493.31, 8776.63}	241.141	0.249621	newly	777.	5048.53	{749., 14 396.}	554.248	0.42287	Reff	2.39443	0.582067	{0.0285303, 1.46295}	75.8763	ar	88 710.	187 054.	{170 009., 245 001.}	110.86
18	R0	{2.02168, 10.1676}	{2.53858, 5.18917}	{{2.00522, 1.29937}, {3., 7.55126}}	{33.1755, 48.9637}	inf	354.	2660.27	{760.645, 2863.22}	656.379	0.615754	newly	351.	3473.	{654., 4429.}	894.667	0.63087	Reff	2.00733	0.498924	{0.141757, 1.11689}	75.1448	ar	80 299.	180 017.	{166 024., 201 185.}	124.184
19	R0	{2.89969, 15.009}	{2.13559, 4.24292}	{{1.05963, 2.64569}, {3., 5.01006}}	{29.5336, 71.7308}	inf	224.	17 538.6	{5212.67, 57 597.7}	7730.53	1.68264	newly	179.	19 088.7	{5446., 56 277.}	10 564.1	1.78777	Reff	2.88972	0.38456	{0.0959919, 0.792709}	86.6921	ar	93 349.	167 828.	{91 795., 268 544.}	86.8302
20	R0	{2.68933, 7.34928}	{2.21748, 9.24499}	{{0.584081, 2.1087}, {3., 15.0628}}	{28.5066, 59.5451}	inf	3.	18 623.5	{6806.22, 36 750.4}	620 682.	3.67834	newly	7.	25 313.5	{4308., 81 809.}	361 521.	3.32768	Reff	2.68909	1.54997	{0.257097, 2.71085}	44.5445	ar	91 574.	118 321.	{55 328., 182 799.}	45.0779
21	R0	{2.15352, 9.28074}	{2.4942, 7.46558}	{{2.15971, 4.3199}, {3., 14.7102}}	{19.0021, 40.4705}	inf	131.	21 003.3	{5920.76, 43 452.4}	15 933.1	2.06198	newly	151.	21 963.1	{3961., 52 159.}	14 445.1	1.97584	Reff	2.14827	0.940398	{0.349782, 2.17121}	56.7535	ar	83 845.	146 640.	{83 259., 212 735.}	76.7002
22	R0	{2.21953, 9.04387}	{2.16716, 6.12541}	{{0.847299, 2.17256}, {3., 10.0955}}	{35.6787, 44.416}	inf	114.	26 397.8	{5667.76, 51 826.9}	23 056.	2.23189	newly	136.	30 977.3	{7901., 58 786.}	22 677.5	2.20936	Reff	2.21565	1.04036	{0.277396, 1.60415}	53.3953	ar	84 856.	125 550.	{57 582., 195 581.}	58.6397
23	R0	{2.21987, 14.5347}	{2.23995, 9.07392}	{{0.415514, 2.711}, {3., 13.7555}}	{41.6772, 37.5706}	inf	1324.	5213.98	{2790.15, 2790.15}	294.473	0.393625	newly	822.	3480.28	{1161., 4021.}	323.392	0.428365	Reff	2.16759	0.36965	{0.0754737, 0.649746}	82.9465	ar	85 067.	178 612.	{170 523., 184 842.}	109.966
24	R0	{2.84268, 17.9659}	{2.16934, 8.42969}	{{0.421116, 2.71738}, {3., 13.7844}}	{29.7746, 53.0795}	inf	799.	3121.68	{2797.75, 2797.75}	292.478	0.55649	newly	439.	2477.71	{1152., 3984.}	466.244	0.682201	Reff	2.80769	0.382275	{0.0757521, 0.650172}	86.3847	ar	92 836.	175 460.	{170 533., 174 886.}	88.9998
25	R0	{2.52706, 10.3679}	{2.0141, 4.73628}	{{0.091794, 0.934266}, {3., 5.83029}}	{38.2651, 58.7889}	inf	97.	3736.02	{591.923, 5479.24}	3751.57	1.38855	newly	97.	4165.64	{486., 6935.}	4194.47	1.35789	Reff	2.52279	0.282827	{0.0134679, 0.451748}	88.7891	ar	89 623.	161 761.	{153 150., 177 467.}	86.7323
26	R0	{2.62835, 19.1088}	{2.44863, 6.67632}	{{1.46292, 3.37346}, {3., 10.2898}}	{22.109, 65.0616}	inf	1699.	4296.25	{1491.91, 8780.71}	176.666	0.215189	newly	814.	4911.22	{753., 13 847.}	509.086	0.396811	Reff	2.55757	0.574089	{0.0287359, 1.46193}	77.5533	ar	90 908.	186 420.	{170 119., 244 805.}	105.065
27	R0	{2.76831, 19.4155}	{2.52748, 4.64903}	{{2.00399, 1.25014}, {3., 7.53083}}	{17.0687, 76.055}	inf	2186.	2338.49	{746.285, 2966.07}	93.6394	0.357496	newly	1084.	3537.93	{686., 5179.}	255.383	0.333418	Reff	2.67358	0.582409	{0.176549, 1.68502}	78.2161	ar	92 175.	183 280.	{165 881., 247 128.}	99.685
28	R0	{2.96609, 16.8903}	{2.47294, 7.93429}	{{2.15947, 4.3072}, {3., 14.7097}}	{17.6109, 53.4397}	inf	591.	22 588.9	{6622.8, 41 095.1}	3722.14	1.45905	newly	383.	22 749.3	{4451., 69 743.}	5839.76	1.59359	Reff	2.94132	0.907045	{0.305371, 2.13478}	69.162	ar	94 024.	150 800.	{83 589., 224 501.}	64.4491
29	R0	{2.17046, 14.39}	{2.24769, 9.26894}	{{0.416433, 2.71125}, {3., 13.7465}}	{43.3178, 35.5876}	inf	1169.	5224.34	{2794.37, 2794.37}	347.267	0.446461	newly	743.	3465.06	{1153., 4068.}	366.361	0.466062	Reff	2.12193	0.372307	{0.0756682, 0.64996}	82.4543	ar	84 001.	178 484.	{170 697., 184 796.}	112.479
30	R0	{2.88772, 12.4647}	{2.26533, 9.38677}	{{0.584228, 2.10867}, {3., 13.2922}}	{25.4671, 33.7658}	inf	43.	18 750.9	{6388.28, 41 164.7}	43 506.8	2.49241	newly	59.	23 726.5	{4246., 70 917.}	40 114.7	2.35125	Reff	2.88584	1.58216	{0.27475, 2.711}	45.7559	ar	93 273.	121 169.	{50 775., 194 867.}	47.8244
31	R0	{2.46831, 8.14308}	{1.72045, 7.89303}	{{0.315933, 2.00079}, {3., 16.4455}}	{42.449, 64.4759}	inf	13.	6944.52	{1101.99, 13 312.5}	53 319.4	2.55992	newly	16.	20 935.8	{2022., 58 690.}	130 749.	2.74394	Reff	2.46792	1.56527	{0.297294, 2.91973}	43.9896	ar	88 762.	67 512.3	{15 912., 105 343.}	37.4005
32	R0	{1.93236, 7.38987}	{2.1845, 5.57538}	{{0.848916, 2.17246}, {3., 10.095}}	{47.4617, 36.6976}	inf	96.	30 889.	{7732.78, 55 384.9}	32 078.4	2.36375	newly	158.	39 972.6	{12 348., 86 692.}	25 201.7	2.2297	Reff	1.92828	1.0902	{0.403936, 1.57476}	46.5859	ar	77 654.	138 705.	{84 560., 206 439.}	79.6494
33	R0	{2.82251, 17.6954}	{2.54458, 4.69468}	{{1.81433, 1.22332}, {3., 12.8073}}	{17.1416, 73.4695}	inf	586.	23 829.4	{4045.15, 59 209.2}	3969.89	1.433	newly	325.	29 368.9	{2380., 73 367.}	8937.85	1.72017	Reff	2.7977	0.629972	{0.25526, 1.13463}	77.4825	ar	92 745.	151 872.	{90 334., 247 903.}	69.1941
34	R0	{1.87083, 12.3994}	{2.26288, 9.03891}	{{0.411775, 2.70364}, {3., 13.7408}}	{54.8502, 37.3748}	inf	1336.	4391.09	{2786.7, 2786.7}	229.324	0.366033	newly	975.	3042.92	{1150., 4007.}	212.094	0.332226	Reff	1.81905	0.377587	{0.074732, 0.651421}	79.2426	ar	76 282.	176 994.	{170 761., 181 418.}	132.026
35	R0	{2.95779, 16.3003}	{2.38234, 4.76043}	{{1.76288, 1.24923}, {3., 12.827}}	{20.7684, 70.7955}	inf	233.	28 541.2	{4882.89, 59 074.3}	12 149.4	1.92578	newly	159.	34 141.8	{4911., 78 663.}	21 372.8	2.12111	Reff	2.94682	0.624535	{0.162345, 1.36593}	78.8065	ar	93 873.	158 548.	{68 922., 258 285.}	79.2651
36	R0	{2.18718, 14.261}	{1.92097, 4.47429}	{{0.0852657, 0.938358}, {3., 5.81945}}	{48.5912, 68.9325}	inf	1258.	5041.76	{551.465, 18 205.9}	321.621	0.4736	newly	857.	5150.02	{477., 16 836.}	517.05	0.575234	Reff	2.13862	0.271314	{0.011763, 0.452998}	87.3136	ar	84 379.	171 102.	{158 025., 227 676.}	105.49
37	R0	{2.44912, 9.31118}	{2.27281, 10.2028}	{{0.858487, 1.99304}, {3., 17.0568}}	{28.6512, 40.8261}	inf	41.	25 014.1	{5314.98, 44 120.2}	60 910.	2.62911	newly	54.	26 370.8	{4032., 61 708.}	48 734.9	2.42586	Reff	2.44728	1.38252	{0.294034, 2.22216}	46.1362	ar	88 442.	128 939.	{82 424., 172 146.}	52.2997
38	R0	{2.05949, 5.72514}	{2.27762, 9.70842}	{{0.763865, 1.99293}, {3., 17.06}}	{37.6471, 87.0633}	inf	11.	25 818.5	{9025.45, 46 839.9}	234 613.	3.24976	newly	26.	28 078.	{5460., 83 383.}	107 892.	2.81062	Reff	2.05842	1.36196	{0.376932, 2.22221}	41.3631	ar	81 587.	128 807.	{79 211., 172 033.}	61.4764
39	R0	{1.75505, 8.52404}	{2.49141, 4.95031}	{{2.00485, 1.28759}, {3., 7.54305}}	{47.9672, 41.9253}	inf	489.	2819.73	{757.506, 2886.71}	480.268	0.490618	newly	574.	3502.15	{674., 7402.}	514.524	0.473615	Reff	1.73539	0.560063	{0.173436, 1.32944}	70.3605	ar	70 925.	182 015.	{166 132., 211 386.}	158.187
40	R0	{2.24724, 13.7861}	{1.8926, 4.52259}	{{0.0852728, 0.938091}, {3., 5.81883}}	{46.3329, 67.7184}	inf	1281.	5743.96	{554.316, 18 323.}	369.474	0.484593	newly	906.	5795.4	{478., 16 907.}	556.954	0.57541	Reff	2.19627	0.28125	{0.00479707, 0.453048}	87.1942	ar	85 257.	170 751.	{157 925., 230 703.}	106.215
41	R0	{1.77635, 8.62406}	{2.0814, 4.36489}	{{1.04887, 2.64624}, {3., 5.00179}}	{49.0052, 50.1339}	inf	634.	13 357.6	{5574.02, 22 492.}	2007.7	1.18676	newly	682.	14 753.3	{6550., 35 289.}	2063.24	1.15556	Reff	1.75212	0.346632	{0.0424633, 0.677055}	80.2164	ar	72 941.	166 816.	{138 773., 226 435.}	133.208
42	R0	{1.80397, 5.4932}	{2.25266, 11.41}	{{1.33132, 6.45725}, {3., 17.0632}}	{44.3172, 118.823}	inf	36.	26 561.2	{5316.32, 57 949.5}	73 681.1	2.67488	newly	83.	23 317.7	{3992., 40 437.}	27 993.6	2.17888	Reff	1.80242	1.3916	{0.288683, 2.22219}	35.7686	ar	73 232.	127 278.	{61 601., 177 719.}	80.3233
43	R0	{2.83096, 16.7077}	{2.42521, 6.75623}	{{1.46529, 3.38759}, {3., 10.3081}}	{20.3038, 59.5622}	inf	491.	2425.8	{1496.7, 6910.09}	395.189	0.570989	newly	311.	3560.98	{774., 6255.}	1045.77	0.734437	Reff	2.80905	0.584943	{0.0597553, 1.40091}	79.1765	ar	92 740.	182 710.	{170 450., 236 364.}	97.0135
44	R0	{2.73678, 11.6021}	{2.24303, 6.139}	{{0.848143, 2.17254}, {3., 10.0958}}	{27.8515, 51.3407}	inf	128.	29 935.2	{7023.76, 61 089.9}	23 288.9	2.21792	newly	125.	37 289.6	{6885., 88 332.}	29 734.8	2.28112	Reff	2.73147	1.03329	{0.257576, 2.17354}	62.1711	ar	92 003.	134 677.	{75 398., 196 415.}	54.964
45	R0	{2.14235, 6.98601}	{2.62338, 7.45145}	{{2.16034, 4.3413}, {3., 14.7085}}	{25.0631, 42.3909}	inf	49.	21 136.2	{5921.16, 35 305.}	43 035.1	2.4774	newly	74.	21 263.1	{3928., 51 851.}	28 633.9	2.27479	Reff	2.14079	0.930476	{0.334434, 2.13116}	57.2332	ar	82 974.	148 967.	{92 567., 218 544.}	82.7844
46	R0	{2.23409, 9.21289}	{2.60136, 7.63873}	{{2.1606, 4.3297}, {3., 14.7052}}	{20.5541, 38.9292}	inf	58.	20 471.3	{5922.02, 38 541.}	35 195.4	2.38526	newly	78.	20 170.1	{4012., 51 684.}	25 759.4	2.22434	Reff	2.23183	0.943178	{0.346944, 2.18538}	57.8533	ar	85 163.	147 240.	{83 228., 213 444.}	76.846
47	R0	{2.12258, 8.15265}	{2.10072, 10.7325}	{{0.498388, 3.60979}, {3., 17.0566}}	{37.9165, 51.3833}	inf	73.	24 175.9	{5316.97, 56 216.6}	33 017.7	2.34611	newly	100.	22 146.8	{4050., 56 541.}	22 046.8	2.09576	Reff	2.11978	1.29166	{0.288743, 2.22216}	42.9773	ar	82 690.	129 177.	{84 594., 174 697.}	61.3775
48	R0	{2.71771, 10.4636}	{2.27944, 10.1795}	{{0.763789, 1.99279}, {3., 17.0586}}	{26.3056, 39.8538}	inf	20.	25 506.9	{5499.22, 46 809.4}	127 435.	2.9492	newly	23.	26 966.5	{4049., 83 287.}	117 146.	2.8026	Reff	2.71654	1.40388	{0.383274, 2.22214}	49.7086	ar	91 716.	131 886.	{77 337., 178 825.}	50.8877
49	R0	{2.44369, 6.66477}	{1.7574, 8.01351}	{{0.372851, 2.00079}, {3., 16.7626}}	{41.8966, 81.5035}	inf	7.	6899.46	{1102.83, 13 522.}	98 463.8	2.82502	newly	14.	21 909.9	{1593., 72 595.}	156 399.	2.7889	Reff	2.44335	1.58365	{0.269689, 2.92653}	43.5705	ar	88 827.	68 746.2	{15 760., 106 672.}	37.0614
50	R0	{2.77383, 17.9775}	{2.43769, 6.65031}	{{1.46392, 3.3779}, {3., 10.2935}}	{20.7612, 63.0076}	inf	1401.	3616.39	{1494.39, 8773.65}	163.38	0.191437	newly	799.	4612.02	{742., 14 075.}	482.884	0.397402	Reff	2.71353	0.571909	{0.0191633, 1.46286}	78.9238	ar	92 334.	185 440.	{170 335., 245 374.}	100.836
